# Retzius-sparing robot-assisted laparoscopic radical prostatectomy: functional and early oncologic results in aggressive and locally advanced prostate cancer

**DOI:** 10.1186/s12894-019-0550-9

**Published:** 2019-11-12

**Authors:** Joanne Nyaboe Nyarangi-Dix, Magdalena Görtz, Georgi Gradinarov, Luisa Hofer, Viktoria Schütz, Claudia Gasch, Jan Philipp Radtke, Markus Hohenfellner

**Affiliations:** 1 Department of Urology, University Hospital Heidelberg, Im Neuenheimer Feld 110, 69120 Heidelberg, Germany; 2 Ruprecht-Karls University of Heidelberg, Medical Faculty, Heidelberg, Germany

**Keywords:** High risk prostate cancer, Retzius-sparing robot-assisted radical prostatectomy, Urinary continence, Erectile function

## Abstract

**Background:**

Retzius-sparing robot-assisted laparoscopic radical prostatectomy (rsRARP) allows entire prostatectomy procedure via the pouch of Douglas. In low- and intermediate-risk prostate cancer (PCa) there is level 1 evidence that the Retzius-sparing approach impacts early continence recovery. Since specific data on aggressive and locally advanced cancer is lacking and avoiding rsRARP is presently suggested, we investigated urinary and sexual recovery, perioperative complications and early oncologic outcomes after rsRARP in this particular cohort.

**Methods:**

Prospectively collected data of 50 consecutive men (median age 66 years) with high-risk PCa who underwent rsRARP in a single institution was analysed retrospectively. The follow-up for all patients was 12 months after surgery.

**Results:**

3 vs. 12 months after surgery, 82% vs. 98% of men used no pad or one safety pad and 50% vs. 72% used no pad. 89% of patients did not observe a decline of continence if postoperative radiotherapy was carried out.

Considering the 17 preoperatively potent patients who underwent bi- or unilateral nerve-sparing surgery, 41% reported their first sexual intercourse within 1 year after rsRARP.

84% of patients had ≥pT3a disease and 42% positive surgical margins. A lymphadenectomy was done in 94% of patients with a median lymph node removal of 15 and lymph node metastasis in 13%.

34% underwent adjuvant radiotherapy and 22% adjuvant androgen deprivation therapy (ADT). 1-year recurrence-free survival was 96%, including 25% of patients on adjuvant or salvage ADT.

**Conclusions:**

RsRARP in high-risk PCa is feasible and results in excellent continence rates, even after postoperative radiotherapy. The potency rates are promising but need further clarification in larger cohorts. Reliable oncologic outcomes require longterm follow-up and are awaited.

## Background

After Binder and Kramer [1] reported the first robot-assisted laparoscopic radical prostatectomy (RARP) in 2001, RARP proceeded to become a widespread and accepted surgical approach for prostatectomy [2]. Meanwhile, the “holy grail” in routine RARP consists of a complete tumor resection and optimal functional results [3]. With oncological outcomes after RARP in localized prostate cancer (PCa) being steadfast, focus is currently shifting towards improvement of perioperative morbidity and functional results.

Reports suggest that surgical techniques involving membranous urethral length preservation [4], posterior musculofascial reconstruction [5] and/or bladder neck preservation [6] may further improve continence. In 2010, Bocciardi et al. reported an innovative posterior Retzius-sparing RARP (rsRARP) approach avoiding bladder detachment [7] thereby presumably minimizing surgical trauma and preserving normal pelvic anatomy maximally. The continence rate reported within the first year of surgery was 96% [8]. In another prospective study, 71% of men after rsRARP were continent 1 week after catheter removal vs. 48% after conventional RARP [9]. These promising results were reported in studies that entirely focused on low- or intermediate-risk PCa collectives. Unknown for this novel technique is whether these functional outcomes can be replicated alongside good oncological outcomes in the challenging setting of high-risk and locally advanced PCa [10].

Men with PCa and a prostate-specific antigen (PSA) > 20 ng/ml, Gleason score 8–10 or clinical-stage ≥T3 are recognized by the major international guidelines to be advanced PCa and constitute the high-risk PCa group [11]. This advanced high-risk-group warrants RP with a clear need for radical/wider dissection, which may compromise functional outcomes [12]. Concerns in these high-risk tumors also regard positive surgical margins (PSMs), inadequate disease control and increased side effects [13]. Recently, accompanied by advances in surgical technology, RP alone or as an initial step within a multimodal concept has gained acceptance in locally advanced PCa [14]. Men with locally advanced PCa treated with RP plus radiotherapy have a lower risk of prostate cancer-specific death compared to men treated with radiotherapy plus androgen deprivation therapy (ADT) alone [15]. EAU guidelines now recognize the role of RP in men with locally advanced PCa, if required in combination with additional treatments such as radiotherapy or ADT [11].

Since rsRARP promotes early continence recovery without an impact on biochemical failure in low- and intermediate-risk PCa, we investigated rsRARP in high-risk PCa. We report the functional, early and midterm oncologic results of our first 50 RARP-patients with high-risk PCa treated by the Retzius-sparing approach.

## Methods

### Patient cohort

This study was a retrospective review of prospectively collected data at the Department of Urology at Heidelberg University Hospital. The study was approved by the ethical committee of the University of Heidelberg (S-403/2012). Our analytic cohort included 50 consecutive patients with aggressive and/or locally advanced PCa undergoing routine rsRARP between July 2016 and September 2017. Men were classified as high-risk when PSA level > 20 ng/ml, Gleason score 8–10 and/or a pathological stage ≥T3 was met, according to recent EAU guidelines [11].

### Surgical technique

All prostatectomies were performed by 3 experienced surgeons routinely performing over 100 RARPs per year. Our rsRARP technique was largely similar to that first described by Galfano et al. [7]. Modifications used in our technique are as described below:

A standard, four-arm da Vinci Si surgical robot was used. Surgeons began with a 0° surgical lens and mostly switched to a 30°-upwards lens beginning at the preparation of the bladder neck henceforth. As a slight technical modification, in contrast to Galfano et al. [7, 8], there was no use of two or four cardinal stitches into the bladder for the identification of the bladder neck orifice during the urethrovesical anastomosis.

### Evaluated clinical and oncological parameters

For each patient, we collected the following clinical and pathologic information: age, preoperative total PSA level, prostate volume, preoperative International Prostate Symptom Score (IPSS), International Index of Erectile Function-5 (IIEF-5) score, biopsy Gleason score, clinical cancer stage (cTNM), prostatectomy Gleason score, pathological cancer stage (pTNM) and PSM rate. Furthermore, the following perioperative variables were evaluated: perioperative transfusion rate, bilateral or unilateral nerve-sparing, intraoperative status of the vesicourethral anastomosis, intraoperative and early postoperative complications, length of hospital stay and time to catheter removal.

Postoperative complications were recorded using the Clavien-Dindo classification [16].

### Follow-up

All patients completed a questionnaire the day before surgery, including the IPSS and the IIEF-5 questionnaire. During the first year after surgery an institutional questionnaire was sent by mail to inquire postoperative complications, the PSA level and the continence and potency recovery. Additionally, patients were interviewed by telephone to complete outstanding information. Minimum follow-up was 12 months.

Patients were considered continent when they used ≤1 safety pad/day. The date on which the patient stopped using more than 1 safety pad was considered the recovery time of continence. Time to recovery of erectile function in preoperatively potent men (IIEF-5 score > 17) was considered the date of the first sexual intercourse after surgery. All men with PSA ≥0.2 ng/ml at analysis were considered biochemically recurrent [11].

### Statistical analysis

Median and interquartile ranges (IQRs) were used to report nonparametric continuous variables. Continence recovery was demonstrated using a Kaplan-Meier plot (IBM SPSS Statistics 24).

## Results

### Perioperative results

Demographic data are summarized in Additional file 1. The median age was 66 years and the median hospitalization was 4 days. The urethrovesical anastomosis was intraoperatively watertight in 92% of patients. A cystogram was performed in all cases on postoperative day 10 to 14. In case of extravasation, a new cystogram was planned. The Foley catheter was removed at a median of 12 days (IQR: 10–14 days).

Complications were classified according to the Clavien-Dindo system. Clavien-Dindo grade I, II and III complications took place in 2, 2 and 6% of cases, respectively. None of the cases required conversion to open surgery. Intraoperative or postoperative blood transfusions were not needed. There was 1 case of rectal perforation which was detected and sutured intraoperatively. Peripheral pulmonary embolism occured in 1 patient. Owed to anastomotic-insufficiency, 1 man was catheterised for 2 months. There were 3 cases of symptomatic lymphoceles, 2 of them requiring percutaneous drainage (Clavien-Dindo grade IIIa) and one requiring laparoscopic lymphocele-marsupialization (Clavien-Dindo grade IIIb).

### Oncologic results

Postoperatively, pathological evaluation revealed 32% with a Gleason score of ≥8 and 84% with extraprostatic extension (pT3-disease). The overall PSM rate was 42% (21 of 50 patients). Eleven patients were identified to have an anterior index tumor in imaging. 8 of 11 (73%) patients with anterior tumors had pT3-disease and 5 of 11 (45%) had PSM. A lymphadenectomy was done in 94% of patients with a median lymph node removal of 15 (IQR 10–21). Lymph node metastasis was documented in 6 of 47 patients (13%).

Postoperative PSA persistence (defined as a PSA level ≥ 0.2 ng/ml at 3 months after surgery) was documented in 5 of 48 (10%) patients. 34% of patients underwent adjuvant radiotherapy and 22% of patients adjuvant ADT.

During 1-year follow-up 2 (4%) patients experienced biochemical recurrence. 2 (4%) patients received salvage treatments: radiotherapy alone or radiotherapy plus ADT. 1-year biochemical disease-free survival with a PSA ≤0.2 ng/ml was 96%. Among them, 12 (25%) patients were on ADT.

### Continence results

**Fig. 1:**
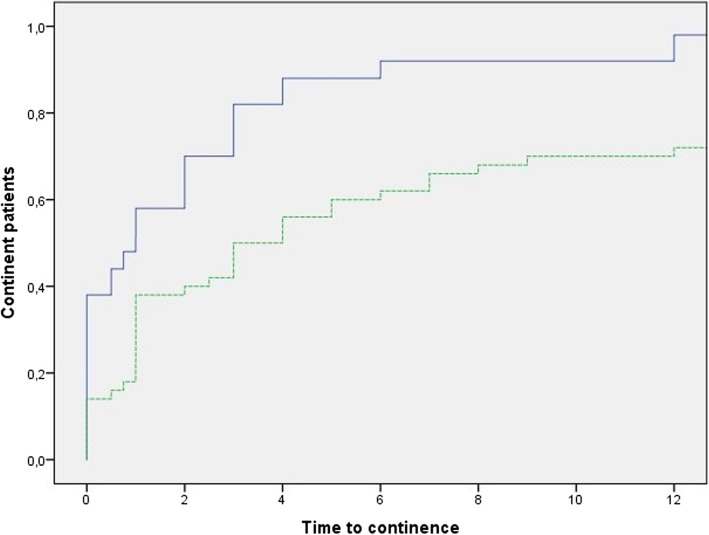
Kaplan-Meier curve of continence recovery. Blue continuous line = no pad or one safety pad; green dashed line = no pad. Time to continence is expressed in months

The urinary continence rate was 38% at 1 week after catheter removal. Three months after surgery, 82% were continent. Figure 1 shows the recovery curve for continence.

Twelve months postoperatively, 98% were continent: while 73% used no pad, 27% used one safety pad per day. One man (2%) used two pads per day. 17 of 19 (89%) who had received adjuvant or salvage radiotherapy observed no decline of continence after radiotherapy. To date, no anastomotic stricture has been detected.

### Erectile function results

Detailed information about sexual function recovery is shown in Additional file 2. 29 (58%) patients were preoperatively potent with an IIEF-5 score > 17. In those patients, bilateral and unilateral nerve-sparing was performed in 12 (41%) and 5 (17%) men, respectively. 7 of the 17 (41%) who underwent bilateral or unilateral nerve-sparing surgery reported to have regular and consistent sexual intercourse within the first year of surgery. 2 of the 7 (29%) postoperatively potent patients used phosphodiesterase type 5 inhibitors on-demand. Median time to recovery of erectile function was 8 months (IQR: 6–11 months). For the 7 postoperatively potent patients, the median IIEF frequency score (Q3) to maintain the erection during sexual intercourse was 4 (IQR: 3–4) and the median IIEF satisfaction score (Q5) was 4 (IQR: 4–5).

## Discussion

Since the Bocciardi group described their technique of a Retzius-sparing approach of RARP in 2010, excellent functional and oncological outcomes have been reported [7, 8]. A recent review found that rsRARP is associated with earlier continence recovery compared to conventional RARP without significant impact on oncological outcomes [17]. Beyond the initiator group, subsequent studies focused on low- and intermediate-risk PCa, leaving a gap in reporting for the high-risk and locally advanced disease cohort [9]. Consequently, due to the lack of data on the outcomes in higher-risk PCa, it has been advised to limit the Retzius-sparing approach to low- or favorable intermediate-risk disease [18]. Since adapting Retzius-sparing prostatectomy in our centre, all men viable for RARP were operated in this manner, regardless of preoperative oncological characteristics. We hereby present the first report that focuses on the perioperative, functional and oncologic results after rsRARP in a small purely aggressive PCa-collective.

Our perioperative results show that Retzius-sparing prostatectomy is not only feasible but safe in aggressive and locally advanced PCa. According to the Clavien-Dindo classification, only Grade III complications or less were detected. Grade III complications occurred in 3 (6%) patients and were due to extended lymphadenectomy yielding up to 40 lymph nodes per patient. Lymphadenectomy is mandatory in this cohort, since it improves the accuracy of cancer staging [19] and may be oncologically beneficial [20]. The lymphocele rate in our study was in accordance with another series reporting occurance in 10% of patients undergoing extended pelvic lymph node dissection [21].

As would be expected in an aggressive and locally advanced collective with PSA level > 20 ng/ml, Gleason score > 7 and/or pathological stage ≥T3, PSMs were higher than in a normal/mixed cohort. In the present analysis, 84% of patients harbored extraprostatic ≥pT3a disease. The present PSM rate (42%) is within range of other reports including a review on high-risk PCa treated with conventional RARP that reported a 16–58% PSM rate [22]. Harty and colleagues performed conventional RARP in a cohort more similar to the present study (high risk and locally advanced PCa). In their cohort with 78% ≥ pT3a disease, 50% had PSMs [23]. An explanation for a reduced PSM rate in rsRARP may be the possibility to dissect antegradely allowing improved visualization of the prostate base and posterior margin of the prostate, which are common sites of PSMs in conventional RARP [24].

Despite knowledge of higher likelihood of PSMs and biochemical recurrence in locally advanced PCa, studies have shown overall survival rates of > 75% at 10 years after RP [22]. Adjuvant therapies following prostatectomy play an important role in surviving locally advanced and high-risk PCa. Reduced biochemical recurrence, distant metastases and longer survival rates of high-risk patients have been repeatedly proved after adjuvant radiotherapy [25]. The role of hormonal manipulation therapy in the high-risk collective is embedded in all national and international guidelines [11]. In our cohort, 34 and 22% of patients underwent adjuvant radiotherapy and adjuvant ADT, respectively. Adjuvant radiotherapy was initiated in men at a high risk of progression according to the histopathological results, e.g. extracapsular extension and positive margins in final pathology specimen results. Adjuvant ADT was initiated in men with lymph node metastasis or with very high-risk localized disease. Twelve months after surgery, 4% of men had received salvage radiotherapy and 2% salvage ADT.

1-year progression-free survival in our cohort was 96%; this included 12 (25%) patients on ADT. In a previously published study on patients with high-risk PCa treated by conventional RARP, the 1-year biochemical recurrence-free survival was 81%, with only 6% primarily scheduled to receive adjuvant treatment [26]. However, current data support the view that overall progression-free survival of high-risk patients is significantly increased when planned adjuvant therapy is administered [25]. It is clear though, that longer oncological follow-up must be awaited.

Defining urinary continence as maximum use of a safety pad per day, 98% of men were continent 12 months after rsRARP in this high-risk cohort. 72% of men were pad free 12 months after rsRARP. Studies of patients with high risk or locally advanced prostate cancer show heterogeneous continence outcomes after RARP. Abdollah et al. analyzed the urinary function of 769 men with high-risk PCa treated with conventional RARP at two high-volume academic centers and revealed urinary continence recovery in 85% of men at 12 months after surgery (defined as the use of no pad or one safety pad per day) [12]. Gandaglia et al. found a 1-year urinary continence recovery rate of 64% after conventional RARP in locally advanced cancer (defined as the use of no pad) [27].

Regarding early continence recovery, 38% of men in our study were continent 1 week after catheter removal and 82% of men 3 months after surgery. With rsRARP in low- and intermediate-risk PCa, early continence was reported in 71% of men 1 week after catheter removal and in 95% of men 3 months after surgery [9]. One explanation for this discrepancy is that our cohort included locally advanced disease with 84% harboring extraprostatic extension in contrast to the previous predominantly pT2-cohort study [9, 28]. The oncological necessity of a more radical and wider dissection plane may explain generally reduced continence rates in high-risk collectives [12]. At the same time, prolonged postoperative catheterisation in the present cohort may have played a vital role. Furthermore, the proneness of high-risk cancer patients to delayed recovery of urinary continence after prostatectomy may also be due to the broader lymphadenectomy template necessary in high-risk PCa; increasing possible damage to nerve fibers innervating the pelvic floor [29]. Nevertheless, Retzius-sparing prostatectomy appears to substantially improve continence rates, rendering a great proportion (84%) of this high-risk cohort, ready for further multimodal therapies like adjuvant radiotherapy.

Men receiving postoperative radiotherapy are known to have a higher overall incontinence rate [30]. Interestingly, only 2 of 19 (11%) patients who received radiotherapy after rsRARP observed a decline in continence after radiotherapy. Previous studies revealed that only 22% of men who are continent at the start of radiotherapy remained continent afterwards [31]. During rsRARP most attachments of the bladder remain untouched and pubourethral and pubovesical attachments remain intact avoiding bladder/urethral hypermobility [32]. This may have a protective effect for continence after radiotherapy. Considering this impact for the adjuvant radiotherapy prone high-risk PCa-group, the Retzius-sparing technique should be investigated in further studies.

For oncological reasons, preservation of erectile function is not highly propagated in men confronted with the diagnosis of high-risk disease [33]. Nonetheless, of the preoperatively potent men with a strong wish for neurovascular bundle preservation and an IIEF-5 score > 17 who underwent unilateral or bilateral nerve-sparing, 41% had had sexual intercourse in the year following rsRARP. Abdollah et al. performed nerve-sparing in 88% of patients with high-risk PCa undergoing conventional RARP and reported that 26% of preoperatively potent men were able to have intercourse 12 months after surgery [12].

Since recovery of sexual function in general continues beyond 12–24 months post surgery [12], it is too early to estimate the full percentage of men that can expect to recover sexual function after rsRARP in high-risk PCa. Neurovascular bundle preservation should, nonetheless, be reserved to highly informed men consenting to nerve-sparing despite increased risk of PSMs in aggressive and locally advanced PCa.

A limitation of this study was that it is non-randomized, non-comparative and retrospective in nature. However, this represents a natural step during the exploration of a new surgical technique. In light of the results of the current study, we initiated a prospective randomized clinical trial at our institution that compares the outcomes of conventional and rsRARP in low-, intermediate and high-risk PCa.

Another limitation was that a single, highly experienced team performed RARP at an academic institution and that the results may not be generalizable.

In addition, it is premature to foresee long-term oncological outcomes in this cohort, since results regarding oncologic efficacy and probability of biochemical failure > 12 months after RARP are pending.

Lastly, the promising first results of sexual function recovery after rsRARP in a purely aggressive PCa-collective need to be confirmed in larger cohorts and over a longer period of time.

## Conclusions

With the increasing role of RARP in the management of aggressive and locally advanced PCa, we present the 1-year functional and oncological outcomes in a cohort of 50 men with high-risk PCa treated with rsRARP. RsRARP is feasible, safe and associated with few perioperative complications. We demonstrate (very) good results regarding the recovery of urinary continence in patients with high-risk PCa. Continence results following rsRARP and postoperative radiotherapy are promising. Potency rates appear encouraging but need further clarification in larger cohorts. Longterm oncological outcomes are awaited.

## Supplementary information

**Additional file 1.** Demographic, preoperative and pathologic features.

**Additional file 2.** Sexual function recovery.

## Data Availability

The datasets used and analysed during the current study are available from the corresponding author on reasonable request.

## Abbreviations

ADT: Androgen deprivation therapy
EAU: European Association of Urology
IIEF-5: International Index of Erectile Function-5
IPSS: International Prostate Symptom Score
IQR: Interquartile range
PCa: Prostate cancer
PSA: Prostate-specific antigen
PSM: Positive surgical margin
RARP: Robot-assisted laparoscopic radical prostatectomy
RP: Radical prostatectomy
rsRARP: Retzius-sparing robot-assisted laparoscopic radical prostatectomy
