# Supplementary material for: Retzius-sparing robot-assisted laparoscopic radical prostatectomy: functional and early oncologic results in aggressive and locally advanced prostate cancer
Source: BMC Urol. 2019 Nov 12;19:113. doi: 10.1186/s12894-019-0550-9 (PMC6852736; doi:10.1186/s12894-019-0550-9)
Supplement: Supplementary file 1 — Additional file 1. Demographic, preoperative and pathologic features. [file 12894_2019_550_MOESM1_ESM.docx]

**Demographic, preoperative and pathologic features**

| **Parameter** | **Quantity** |
| --- | --- |
| Age, yr, median (IQR) | 66 (62-71) |
| PSA level, ng/ml, median (IQR) | 8,9 (5,2-16,7) |
| Gleason score at biopsy (%) |  |
| 7 | 34 (68) |
| 8 | 5 (10) |
| 9 | 11 (22) |
| Suspicious digital rectal examination, ≥T2 (%) | 25 (50) |
| Prostate volume, median (IQR) | 36 (26-47) |
| Length of hospital stay, days, median (IQR) | 4 (3-4) |
| Length of catheterization, days, median (IQR) | 12 (10-14) |
| Pathologic Gleason score (%) |  |
| 7 | 34 (68) |
| 8 | 4 (8) |
| 9 | 12 (24) |
| Pathologic T stage (%) |  |
| pT2 | 8 (16) |
| pT3a | 28 (56) |
| pT3b | 14 (28) |
| Pelvic lymph node dissection (%) | 47 (94) |
| Number of removed lymph nodes, median (IQR) | 15 (10-21) |
| Lymph node-positive patients on pathology (%) | 6 (13) |
| Number of positive lymph nodes, median (IQR) | 2 (1-3) |
| Positive surgical margins |  |
| Overall (%) | 21 (42) |
| Multifocal (%) | 12 (57) |
| Unifocal (%) | 9 (43) |

IQR = interquartile range; PSA = prostate-specific antigen; yr = year
