# Supplementary material for: Retzius-sparing robot-assisted laparoscopic radical prostatectomy: functional and early oncologic results in aggressive and locally advanced prostate cancer
Source: BMC Urol. 2019 Nov 12;19:113. doi: 10.1186/s12894-019-0550-9 (PMC6852736; doi:10.1186/s12894-019-0550-9)
Supplement: Supplementary file 2 — Additional file 2. Sexual function recovery. [file 12894_2019_550_MOESM2_ESM.docx]

**Sexual function recovery**

A

| **Patients (n)** | **Postop. IIEF ≥22** | **Postop. IIEF 16-21** |  | **Postop. IIEF 0-15** |  |
| --- | --- | --- | --- | --- | --- |
| **Preop. IIEF ≥22** | 7 | 4 |  | 9 |  |
| **Preop. IIEF 16-21** | 0 | 1 |  | 12 |  |
| **Preop. IIEF 0-15** | 0 | 1 |  | 16 |  |

IIEF = International Index of Erectile Function-5; postop. = postoperative; preop. = preoperative.

The 50 men of the cohort were preoperatively separated into potent (IIEF ≥22), with erectile dysfunction (IIEF 16-21) and impotent (IIEF 0-15). 12 months postoperatively, the IIEF scores were re-examined. The number of patients in each category is given.

B

| **Patients (n)** | **Postop. IIEF ≥22** | **Postop. IIEF 16-21** | **Postop. IIEF 0-15** | **On ADT** |
| --- | --- | --- | --- | --- |
| **Preop. IIEF >17** | 7 | 5 | 14 | 3 |

ADT = androgen deprivation therapy; IIEF = International Index of Erectile Function-5; postop. = postoperative; preop. = preoperative.

The 29 patients with preoperative IIEF >17 were separated into potent (IIEF ≥22), with erectile dysfunction (IIEF 16-21), impotent (IIEF 0-15) and on ADT at 12 months postoperatively. The number of patients in each category is given.
